# Features, Design, and Adherence to Evidence-Based Behavioral Parenting Principles in Commercial mHealth Parenting Apps: Systematic Review

**DOI:** 10.2196/43626

**Published:** 2023-06-01

**Authors:** Kexin Li, Katherine I Magnuson, Grace Beuley, Logan Davis, Stacy R Ryan-Pettes

**Affiliations:** 1 Department of Psychology and Neuroscience Baylor University Waco, TX United States

**Keywords:** mobile phone, parent, behavioral parent training, parent management training, mobile apps, mobile health, mHealth, child, adolescent

## Abstract

**Background:**

There is a need to disseminate evidence-based parenting interventions for adolescent externalizing concerns. Although family-based treatments have demonstrated efficacy for such concerns, they have limitations and challenges when disseminated in the community. Behavioral-based parenting techniques form an integral part of well-established, family-based interventions for adolescent behavioral problems and are ideal for dissemination through coupling with smartphone technology. Despite the vast number of “parent” apps currently available in commercial markets, there is a dearth of reviews focused on evaluating mobile health apps through the lens of behavioral parenting training (BPT).

**Objective:**

This study aimed to conduct a systematic review of commercial mobile health apps for parents to increase effective parenting skills that include behavioral components.

**Methods:**

A search of the Google Play and Apple App Stores identified 57 apps that were included in the review and coded for availability, popularity, and infrastructure. In total, 89% (51/57) of them were sufficiently functional to be assessed for app design quality (engagement, functionality, esthetics, and information), and 53% (30/57) proceeded to the final evaluation of level of adherence to BPT principles.

**Results:**

In total, 57 apps met the initial inclusion criteria. Accessibility was high across these apps given that 44% (25/57) were available on both the Google Play and Apple App Stores and 68% (39/57) were free of charge. However, privacy concerns were addressed inconsistently among the apps. App design quality was average across the included apps, and apps with positive user star ratings or a high number of downloads received higher ratings on app design quality. In contrast, the identified apps largely fell short in providing BPT components adequately and with high interactivity, with low levels of adherence to BPT (mean 20.74%, SD 11%) across all commercial apps evaluated. Commercially popular apps did not show higher levels of adherence to BPT. Overall, a moderate relationship between app design quality and adherence to BPT was found. App features that have been found to increase user engagement, such as gamification and individualization, were only observed in a small minority of apps. Overall, there was a lack of focus on teenage development.

**Conclusions:**

Future app developers hoping to increase the dissemination of BPT should aim for free and accessible apps that balance high-quality design features (eg, simple esthetics, interactivity, and individualization) with content consistent with BPT principles. They should also consider key issues that are inconsistently addressed in current apps, including privacy and teenage development. Future app developments will likely benefit from multisector (industry and academic) collaboration throughout the design process and involving end users (ie, parents) during different stages of app development.

## Introduction

### Background

The need for effective parenting interventions for parents of adolescents with behavioral problems in community service settings is an important public health issue in the United States [1,2]. National surveys indicate a high prevalence of behavioral or conduct problems in children and adolescents, whereas only half have received any treatment [3]. Despite this high demand, there is a substantial lack of evidence-based parenting support programs specific to parents or as part of services for adolescents in the community [1]. Although family-based therapies are more commonly found in community mental health clinics and are efficacious in increasing the use of effective parenting strategies [4,5], barriers such as cost and provider expertise limit implementation and dissemination [1]. However, behavioral-based parenting techniques [6-8] are an integral part of these interventions [9,10] and can be effectively delivered through smartphone technology [11].

Mobile health (mHealth) has emerged as a promising option for using smartphone technology to increase the reach of behavioral-based parenting programs such as behavioral parenting training (BPT) for parents of adolescents with behavioral problems [12]. The commercial industry of mHealth has certainly proliferated, and there is parent demand. The number of health and wellness app downloads has reached an estimated 3.35 billion worldwide [13], and previous research suggests that parents turn to resources on the internet or smartphone apps to receive help with parenting to manage child behaviors [14,15]. Moreover, parents of teenagers with behavioral problems such as substance misuse have expressed interest in receiving support delivered through mHealth [16]. Although there has been a proliferation of commercial mHealth apps, research has not kept pace with evaluating parenting apps through the BPT framework [14,17,18], which limits our understanding of the usefulness of commercially available parenting apps for the management of child or adolescent behavioral problems.

The goal of this review was to identify commercial mHealth apps that include components of behavioral parenting techniques and evaluate the identified apps on app features, app design, and adherence to the theoretical framework behind BPT techniques and interventions. The intent of this review was to inform the selection of mHealth apps by providers and the development of new apps designed for parents of adolescents with behavioral problems [17].

Although there are several systematic reviews evaluating commercial mHealth apps related to physical activity (eg, exercise) and medical health conditions (eg, diabetes) [19], there are fewer reviews of commercial mHealth apps for mental health interventions other than for adult depression and substance misuse [20,21]. Regarding commercially available parent-targeted mHealth apps, the vast majority of previous reviews have focused on evaluating apps that provide information or interventions for medical or health concerns such as pregnancy; prenatal, postnatal, and infant care [18,22-28]; nutrition, physical activity, weight management, and medication adherence [29-31]; or parent education and health literacy about particular topics [14,32]. In comparison, few reviews of commercial apps have focused on evaluating the content and quality of parenting apps devoted to providing skills to parents through a BPT lens [17] (Magnuson, PsyD, unpublished data, May 2023).

We found 2 systematic reviews of parent-targeted commercial mHealth apps that focused on parenting children who have behavioral or mental health concerns [33,34]. Păsărelu et al [34] reviewed mobile apps addressing parenting skills for the management of child attention-deficit/hyperactivity disorder and noted the presence or absence of evidence-based elements. However, the review did not comprehensively evaluate the recommended treatments covered in the apps. Trahan et al [33] focused on parenting apps designed for low-income fathers in the United States but included a special focus on evaluating the apps through the lens of paternal self-efficacy and evidence-based factors that contribute to low-income father engagement. Evaluation of the extent to which apps adhere to specific evidence-based intervention strategies or theoretical principles is needed in a review of commercial mHealth apps for parenting practices.

### Objectives

Given the empirical basis of BPT for child behavioral problems, the potential advantages of leveraging mHealth apps to deliver BPT, and the dearth of reviews focused on evaluating mHealth apps for their BPT or BPT-informed components, the major objective of this study was to conduct a systematic review of commercial mHealth apps for parents to increase effective parenting skills that include behavioral components. More specifically, we aimed to answer four main research questions regarding the identified apps: (1) What are the general characteristics of parent-targeted apps with BPT components or BPT-informed components that are available in the most frequently used commercial app stores (ie, Apple App Store and Google Play Store)? (2) What is the app quality (using the Mobile App Rating Scale [MARS] framework [35]) of parent-targeted apps with BPT components or BPT-informed components? (3) Are commercial apps with BPT components or BPT-informed components adherent to the theoretical framework of behavioral parenting interventions? and (4) What is the association among app characteristics (eg, platform and popularity), design quality, and adherence to the theoretical framework of BPT?

The results are discussed within the context of optimizing the digital translation of BPT into mHealth apps for parents. This review provides a picture of what is on the menu for the general public and could inform future development of mHealth apps that aim to provide behavioral parenting interventions for parents of children with behavioral problems.

## Methods

### Search Strategy

A systematic review framework was used to search, screen, and assess commercial apps in English. An initial search of the US Google Play and Apple or iOS App Stores was completed by the first author (KL) in December 2021. A second, independent, parallel search of both stores was also conducted by the third author (LD) to ensure reproducibility and confirm the comprehensiveness of the initial app search. The searches focused on the US Google Play and Apple App Stores as they are the 2 leading mobile phone app markets [36,37]. Search terms were selected based on type of intervention (ie, parenting), target audience (ie, parents), and topic of concern (ie, child externalizing or behavioral concerns), consistent with the goals of this review. Colloquial paraphrases were added based on descriptions in app stores and terms used in previous reviews. Specific search terms included “parent,” “parenting,” “parenting tips,” “parenting advice,” “parenting teens,” “child behavior,” and “child discipline.”

The preliminary determination of inclusion and exclusion was conducted based on descriptions and images published by app developers on the US Google Play and Apple App Stores, a process consistent with previous commercial app reviews [38]. Apps with questionable eligibility were downloaded for further analysis, and those that did not meet the eligibility criteria were not included in this review.

Apps that met the inclusion criteria were downloaded and installed on an iPhone XS or Samsung Galaxy S10 Plus (SM-G975U) device depending on the accessibility of each app in either system. Apps were then assessed based on four broad domains: (1) accessibility and popularity, (2) infrastructure, (3) app quality, and (4) adherence to behavioral parenting principles or strategies. Apps that were unusable or rated as having low interactivity under app quality (see the *Data Extraction* section) were excluded from further evaluation of BPT adherence.

### Inclusion and Exclusion Criteria

This study included commercially available apps whose primary function is to offer parenting skills or “tips” to address child or adolescent behaviors that are key targets for behavioral parenting techniques such as co-operation; compliance; or establishing expectations, rules, and limits (eg, completing chores).

This study excluded apps that (1) were not offered in English; (2) were intended for use by professionals or individuals other than parents or caregivers; (3) required access codes from a health professional or a research or medical program to be used; (4) targeted areas of parenting or parent education unrelated to managing child behaviors (eg, pregnancy or infant care, information on developmental milestones, and other medical or nutritional needs of children); (5) were aimed at infant or prechildhood care and milestone trackers; (6) were listed for “age 0 to 3” (apps listed for “age 0 to 5” were further evaluated for whether they contained BPT components); (7) were intended for coparenting because of this review's focus on improving parenting in parent-child contexts; (8) functioned as communication platforms between parents and care professionals (eg, health care professionals, pediatricians, and childcare or daycare options) or schools; (9) focused on early learning, homeschooling, or games for cognitive development; (10) were designed for specialized developmental or neurodevelopmental conditions (eg, autism spectrum disorder, developmental delays, learning disorders, and special education or needs) as these concerns often require additional interventions and approaches [34,39] distinct from parenting techniques that may be part of the intervention [40]; (11) functioned as catalogs, electronic libraries, or electronic books or magazines for parenting; (12) functioned as forums or internet-based communities; or (13) functioned exclusively as technical aids to facilitate implementation of specific parenting activities (eg, token or reward trackers, house chore checklists, digital safety monitors, or geographic locators) without providing interventions or teaching underlying BPT principles such as reinforcement, house rules, and monitoring and supervision. In contrast, apps that included embedded technical aids (eg, token trackers) but also provided some verbal guides on practicing related parenting skills were included in this review.

### Data Extraction

Retrieved apps were categorized and evaluated based on the criteria used to evaluate commercial apps across previous reviews [36,37], including accessibility and popularity, infrastructure, app quality, and level of adherence to BPT strategies and their underlying principles [41,42].

#### Accessibility and Popularity

Accessibility was coded based on whether the app could be found on one or both app stores and on cost. Given that the actual number of views or downloads of an app is rarely accurately reported on either the Google Play or Apple App Store [36], several indicators of popularity were coded as approximations. These indicators included any available information provided about the number of downloads, number of users that rated the app, and average user star ratings (based on a 5-star system). However, it should be noted that, typically, only apps with a substantial pool of user ratings will display their specific number of reviews and average star ratings.

#### Infrastructure

To evaluate app infrastructure, the evaluators coded whether the app had a privacy statement and, if so, where one could access it (ie, on the app store, on a separate website, or within the app). In addition, the evaluators explored whether there was a website associated with each app as an indicator of the app’s supporting infrastructure.

#### App Quality

The MARS was used to evaluate app quality. The MARS is a 23-item scale designed to provide objective and reliable multidimensional measures of the quality of health-related apps [35]. It was developed by a multidisciplinary team of psychologists, scientists, and technology development experts. It encompasses several subscales, including engagement (eg, interactivity), functionality (eg, ease of use), esthetics (eg, graphics), and information quality and effectiveness (eg, accuracy of app description on the app store) and has emerged as a promising measure of user experience and quality of health apps [35]. The rating of each MARS item is based on a 5-point Likert scale (1=inadequate, 2=poor, 3=acceptable, 4=good, and 5=excellent). Previous research has shown that the MARS has strong internal consistency (Cronbach α=.92) and interrater reliability (intraclass correlation coefficient [ICC]=0.85) [35]. For this study, the internal consistency of the primary evaluator (KL) was acceptable (Cronbach α=.79). To calculate reliability, a second independent rater (LD) randomly chose 20% of the included apps (10 apps) to rate app quality, and the interrater reliability between the 2 evaluators was moderate (ICC=0.52) [43].

#### Adherence to Behavioral Parenting Strategies and Principles

Adherence to the strategies and principles of behavioral parenting interventions was evaluated by examining app content against 91 BPT strategies that are part of the most widely used protocols. The strategies were distilled from current protocols following a sequence of steps by the first (KL) and second (KM) authors, who are doctoral clinical psychology students with training in cognitive and behavioral theories and child interventions related to BPT. A codebook that listed the behavioral parenting strategies was developed to guide our codes. For this study, the core clinical strategies for BPT were summarized as a list of individual statements comprising the codebook by the first author (KL).

Specifically, first, the first (KL) and second (KM) authors and a team of trained undergraduate students searched the scholarly literature, treatment manuals, and nonacademic web-based sources promoting effective parenting strategies in managing child or adolescent behaviors. The first and second authors reviewed the comprehensive compilation and generated a list of parenting strategies grouped by domain. This process of search and consolidation was supervised by the senior author (SRP)—a licensed psychologist specializing in treating child and adolescent externalizing behavior. In addition, the first author independently consulted several evidence-based treatment manuals grounded in BPT and social learning theory as well as manuscripts on behavioral parenting mechanisms of change [44,45] to add to or revise the list of strategies and domains for the final list of BPT strategies. The manuals that were reviewed included the Oregon model of parent management [8,46-48], the behavior modification approach to parenting by Kazdin [6], Helping the Noncompliant Child [9], Parent-Child Interaction Therapy [41], Functional Family Therapy [42,44], and Multisystemic Family Therapy [10].

Second, a codebook that listed the behavioral parenting strategies was developed to guide data extraction while reviewing the commercial apps. For this study, the core clinical strategies for BPT were summarized as a list of individual statements by the first author (KL). A total of 91 unique core statements or BPT strategies were identified and grouped into twelve domains: (1) psychoeducation (2 statements), (2) setting behavioral targets and goals (6 statements), (3) tracking (9 statements), (4) supervision (5 statements), (5) positive reinforcement (15 statements), (6) positive verbal support or praise (6 statements), (7) consequences (15 statements), (8) setting limits or clear rules (11 statements), (9) making effective requests (9 statements), (10) communication and family relationship (7 statements), (11) parent mental health and self-care (3 statements), and (12) maintenance and additional resources (3 statements). See Multimedia Appendix 1 for the complete list of 91 core statements grouped by domain.

Third, consistent with previous reviews evaluating adherence to evidence-based principles [36,45], the first author (KL) rated apps against each of the 91 core statements on a scale from 0 to 2 to evaluate adherence, where 0=there was no information about the statement, 1=there was some information related to the core statement (eg, information was incomplete or implicit), and 2=the app provided explicit and comprehensive verbal instruction that paraphrased the statement. After each core statement was rated, the mean adherence score of each domain was obtained by averaging the ratings of all statements within this domain. Finally, an overall adherence level was obtained by dividing the sum of the domain scores by the maximum possible total score of 24 (ie, 2 points for each of the 12 domains) and converting it to a percentage. A percentage was calculated instead of the mean score to reflect a more diverse range of possible adherence levels. Table 1 displays an illustration of the scoring system using the psychoeducation and setting behavioral targets and goals domains as examples. The remaining 10 domains were scored following the same procedure.

Only apps scoring ≥3 on the MARS interactivity evaluation were coded for adherence to behavioral parenting strategies and principles. The apps that were not evaluated for adherence were those with potentially limited impact on changing user behaviors. Previous research indicates that interactivity (defined in the MARS as whether the app “allows user input, provides feedback, contains prompts such as reminders, sharing options, notifications, etc” [35]) is a key consideration for effectively translating traditional face-to-face behavioral health interventions to digital platforms [49-51]. Interactivity considerably affects the user’s attitude toward the digital intervention, intentions for behavior change, and the manner and extent to which intervention content is psychologically processed by the user [52-54]. Low interactivity likely substantially limits the effectiveness of behavior change interventions delivered digitally [54-56]. Consistent with this literature, those apps that scored either 1 or 2 on the item “interactivity” (indicating below-average or below-sufficient interactivity) based on the MARS evaluation were excluded from the evaluation of adherence because of the potentially limited effect in changing user behaviors.

In this study, the internal consistency of BPT adherence for the primary evaluator (KL) was acceptable (Cronbach α=.75). To calculate reliability, a second independent rater (LD) randomly chose 20% of the final apps (7 apps) to rate adherence to BPT principles. The interrater reliability between the 2 evaluators was moderate (ICC=0.63) [43].

**Table 1:** Scoring system for each app using 2 domains as an illustration.

Domain and statement			Rating for each statement							Domain mean adherence (range 0-2)			Overall adherence (range 0%-100%)
			0		1		2						
**Psychoeducation**													
	Normative child development for child’s age	None		Some		Clear		Average rating of all items in psychoeducation domain			Sum of all domain means divided by the total score of 24 (ie, 2 points × 12 domains), converted to a percentage		
	Problem or risk behaviors for child’s age	None		Some		Clear		Average rating of all items in psychoeducation domain			Sum of all domain means divided by the total score of 24 (ie, 2 points × 12 domains), converted to a percentage		
**Setting behavioral targets and goals**													
	Goals are achievable, realistic, developmentally appropriate, measurable, specific, and time limited	None		Some		Clear		Average rating of all items in the setting behavioral targets and goals domain			Sum of all domain means divided by the total score of 24 (ie, 2 points × 12 domains), converted to a percentage		
	Selection of behaviors to target	None		Some		Clear		Average rating of all items in the setting behavioral targets and goals domain			Sum of all domain means divided by the total score of 24 (ie, 2 points × 12 domains), converted to a percentage		
	Defining problem behavior as well as replacement behavior	None		Some		Clear		Average rating of all items in the setting behavioral targets and goals domain			Sum of all domain means divided by the total score of 24 (ie, 2 points × 12 domains), converted to a percentage		
	Pinpointing	None		Some		Clear		Average rating of all items in the setting behavioral targets and goals domain			Sum of all domain means divided by the total score of 24 (ie, 2 points × 12 domains), converted to a percentage		
	Guidance on establishing behavior contract	None		Some		Clear		Average rating of all items in the setting behavioral targets and goals domain			Sum of all domain means divided by the total score of 24 (ie, 2 points × 12 domains), converted to a percentage		
	Guidance on establishing token economy	None		Some		Clear		Average rating of all items in the setting behavioral targets and goals domain			Sum of all domain means divided by the total score of 24 (ie, 2 points × 12 domains), converted to a percentage		

### Statistical Analysis

Data were collected by manually coding the extracted data, and all statistical analyses were performed using SPSS (version 28; IBM Corp [57]). The relevant characteristics of accessibility (eg, cost and platform), popularity, and infrastructure were determined in advance. Summary statistics, including means, SDs, and percentages, were used to describe app characteristics of interest. Descriptive statistics were used to describe the quality of the apps based on the MARS subscales and adherence to BPT strategies and principles across the 12 domains. Independent-sample 2-tailed *t* tests and 1-way ANOVAs were performed on MARS scores and BPT adherence scores between apps with different characteristics (eg, platform and popularity). The relationships between the MARS ratings and BPT adherence scores were computed using Pearson *r* correlations.

## Results

### Search of mHealth Apps

The search of the commercial marketplace identified a total of 1012 apps (657/1012, 64.92% from the Google Play Store and n=355, 35.08% from the Apple App Store). After duplicates (88/1012, 8.7%) were removed, the search yielded 924 unique apps related to “parenting.” After exclusion, 57 apps met the final criteria for inclusion in the review (Multimedia Appendix 2). Notably, a substantial number of apps (87/924, 9%) were excluded because they only served as digital aids or tools that assisted the implementation of specific behavioral parenting techniques (eg, token tracker and house chore checklist) but did not provide information on how to practice the skills.

**Figure 1:**
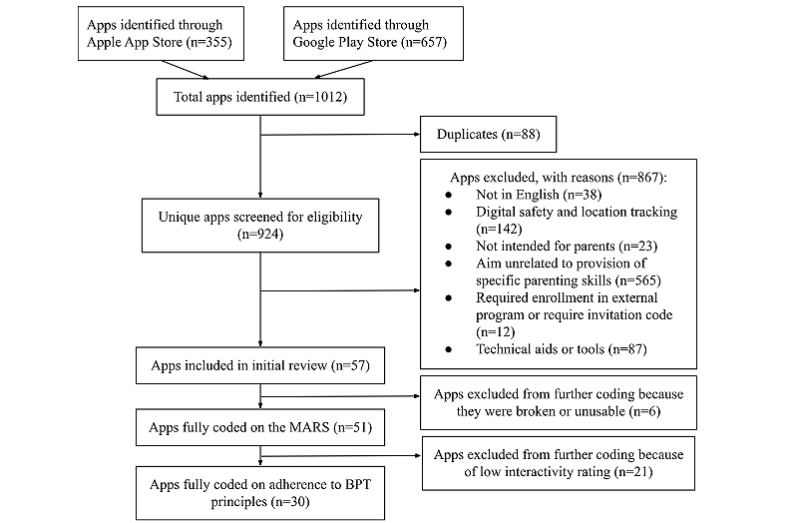
Flow diagram illustrating the exclusion of apps at various stages of the study. BPT: behavioral parenting training; MARS: Mobile App Rating Scale.

After excluding 11% (6/57) of the apps, which were unusable owing to technological glitches within the app, a total of 51 apps had complete MARS ratings. An additional 21 of the 57 (37%) apps were excluded due to receiving low interactivity rating on MARS. These excluded apps primarily presented parenting skills in a text-based way that could be similarly achieved by reading a book or through a web-based catalog search (eg, presenting chapters of books and listing web-based articles related to parenting). After exclusion of low-interactivity apps, 30 apps were analyzed for adherence to behavioral parenting principles (Figure 1).

### App Characteristics

#### Accessibility

Of the 57 apps included in the review, 24 (42%) were only available on the Google Play Store, 8 (14%) were only available on the Apple App Store, and 25 (44%) had versions on both platforms. Consistent with previous research [36,37], there appeared to be more apps in general through the Google Play Store compared with the Apple App Store. Of the 57 apps included, 49 (86%) were free to download. Of the 8 paid apps, 1 (12%) cost US $0.99, a total of 5 (62%) cost US $2.99, a total of 1 (12%) cost US $3.99, and 1 (12%) cost US $9.99 to download (mean US $3.74, SD US $2.49). Of the 49 apps that were free to download, 10 (20%) involved in-app purchases or subscriptions. In-app costs depended on whether the user selected a monthly or yearly subscription, ranging from US $0.99 to US $14.99 for the initial period of subscription (eg, 1 month).

#### Popularity

For each app, the Google Play Store reported a range of the number of downloads to date, whereas the Apple App Store did not. On the basis of the 49 apps listed on the Google Play Store, the median number of downloads was between 1000 and 5000. A total of 10% (5/49) of these apps were installed <100 times. Across both the Google Play and Apple App Stores, average star ratings were only reported for apps with user downloads or sufficient user star ratings. Among the 41% (20/49) of apps that reported user ratings on the Google Play Store, the mean number of ratings was 610.55 (SD 2138.76; range 2-9892), and the mean star rating across the apps was 4.05 (SD 0.72; range 2.7-5) out of 5. In the Apple App Store, 48% (16/33) of apps reported user ratings. The mean number of ratings was 114.81 (SD 226.63; range 1-734), and the mean rating was 4.52 (SD 0.74; range 2-5) out of 5.

A total of 40% (23/57) of unique apps were considered highly rated by users based on an average user rating of >3 on either the Google Play or Apple App Store (Multimedia Appendix 2). The use of 3 as the cutoff was consistent with previous reviews of commercial apps [21].

Most user star ratings and numbers of downloads centered on the same 4 apps (The Happy Child, Weldon, Parent Lab, and Thumsters), all of which had versions on both platforms. On the basis of the available information on the Google Play Store, 75% (3/4) of these apps had >10,000 downloads, whereas Thumsters had between 1000 and 5000 downloads.

#### Infrastructure

Of the 57 apps included in this review, 18 (32%) did not have their own associated website. In terms of the location of the privacy policy, 2% (1/57) of the apps had their privacy policy located exclusively within the app. A total of 32% (18/57) of the apps did not have any privacy policy (including 1/57, 2% of apps whose website was unreachable), 58% (33/57) provided privacy policies that were accessible within the web-based app store, and the remaining 9% (5/57) provided privacy policies on websites external to the app store.

### App Design Quality

#### Overall Quality

A total of 89% (51/57) of the apps were evaluated for app quality using the MARS as 11% (6/57) were unable to function correctly and, thus, could not be evaluated further (Figure 1). See Multimedia Appendix 3 for the MARS ratings of each app. The overall objective quality of all 51 apps was rated as average (mean 3.86, SD 0.51). Regarding the subscales, the apps received the highest rating on functionality (mean 4.50, SD 0.40), followed by esthetics (mean 3.80, SD 0.78), information (mean 3.63, SD 0.64), and engagement (mean 3.49, SD 0.72).

#### App Quality Between Platforms

A 1-way ANOVA was conducted to examine whether the MARS score was related to being on one or both platforms (Table 2). Significant differences were found in the overall MARS score as well as in the engagement, esthetics, and information subscales at *P*<.01. The results consistently showed that apps available on both platforms received significantly higher overall score, higher engagement, and higher esthetics at *P*<.001 and higher information at *P*=.004, compared to apps exclusive to the Google Play Store. However, there was no statistically significant difference between apps exclusive to the Apple App Store and apps found on both platforms or apps exclusive to the Google Play Store. Notably, there was no significant difference found between platforms in the functionality subscale (*P*=.49), which suggests that apps had similar levels of functioning, performance, and usability across platforms.

**Table 2:** Mobile App Rating Scale (MARS) scores by platform (n=51).

MARS	Apple App Store (n=6), mean (SD)	Google Play Store (n=22), mean (SD)	Both (n=23), mean (SD)	*F* test (*df*)	*P* value (2-tailed)
Overall	3.91 (0.31)	3.54^a^ (0.47)	4.14^a^ (0.41)	11.27 (2,48)^b^	<.001
Engagement	3.57 (0.48)	3.05^a^ (0.67)	3.89^a^ (0.57)	10.91 (2,48)^b^	<.001
Functionality	4.67 (0.13)	4.5 (0.39)	4.45 (0.45)	0.73 (2,48)	.49
Esthetics	3.72 (0.61)	3.29^a^ (0.71)	4.30^a^ (0.53)	14.88 (2,48)^b^	<.001
Information	3.70 (0.52)	3.31^a^ (0.57)	3.92^a^ (0.61)	6.133 (2,48)^b^	.004

^a^Means differ from each other at *P*<.001 (Tukey honestly significant difference [HSD] test).

^b^*P*<.01.

#### App Quality Based on User Star Ratings

Independent-sample 2-tailed *t* tests were used to determine whether there was a difference in MARS scores between apps with user star ratings of >3 and apps with low or no user ratings (Table 3). There were significant differences in overall MARS, engagement, esthetics, and information scores. Compared with apps with low or no user ratings, apps with higher user ratings received significantly higher overall MARS (*P*=.008), engagement (*P*=.01), esthetics (*P*=.01), and information (*P*=.03) scores. There was no significant difference in the functionality subscale between the 2 groups (*P*=.67).

**Table 3:** Mobile App Rating Scale (MARS) scores of highly rated apps compared with others (n=51).

MARS	High rating (n=23), mean (SD)	Low or no rating (n=28), mean (SD)	*t* test (*df*)	*P* value (2-tailed)
Overall	4.06 (0.46)	3.69 (0.49)	2.75 (49)	.008
Engagement	3.76 (0.65)	3.26 (0.70)	2.58 (49)	.01
Functionality	4.52 (0.35)	4.47 (0.44)	0.43 (49)	.67
Esthetics	4.10 (0.73)	3.55 (0.74)	2.67 (49)	.01
Information	3.85 (0.50)	3.46 (0.71)	2.21 (49)	.03

#### App Quality of the 4 Most Downloaded Apps—The Happy Child, Weldon, Parent Lab, and Thumsters

Independent-sample 2-tailed *t* tests were used to determine whether there was a difference in MARS scores between the 4 most downloaded apps and the remaining apps (Table 4). Statistically significant differences were found across all comparisons (ie, overall, *P*=.002; engagement, *P*=.007; functionality, *P*=.05; esthetics, *P*=.01; and information, *P*=.04), with the 4 most downloaded apps consistently receiving higher average MARS scores than the remaining apps. The largest differences were observed in the overall MARS score and the engagement and esthetics subscales.

**Table 4:** Mobile App Rating Scale (MARS) scores of the most downloaded apps compared with others (n=51).

MARS	Most downloaded (n=4), mean (SD)	Others (n=47), mean (SD)	*t* test (*df*)	*P* value (2-tailed)
Overall	4.57 (0.19)	3.79 (0.48)	3.2 (49)1	.002
Engagement	4.4 (0.26)	3.41 (0.69)	2.84 (49)	.007
Functionality	4.88 (0.14)	4.46 (0.40)	2.05 (49)	.046
Esthetics	4.75 (0.33)	3.71 (0.75)	2.7 (49)	.01
Information	4.27 (0.39)	3.58 (0.63)	2.11 (49)	.04

### Adherence to Behavioral Parenting Principles

#### Overall and Domain-Specific Adherence

Across all 30 apps (after removing apps with low interactivity), overall adherence to BPT principles was quite low (mean 20.74%, SD 11%). This is consistent with previous reviews of commercial apps that examined adherence between mental health apps and treatment principles (eg, mHealth apps for depression and cognitive behavioral therapy principles) [36,37]. The median level of adherence to BPT principles was 19.30% (range 5.56%-44.69%). See Multimedia Appendix 4 for the BPT adherence ratings for each app.

The relationship and communication domains had the highest average adherence and were addressed by most of the apps (27/30, 90%). In contrast, supervision had the lowest mean level of adherence and was addressed by only 13% (4/30) of the apps. In addition, none of these 13% (4/30) of apps that addressed supervision received an adherence rating of >1. Table 5 shows the number of apps that addressed each domain and the mean levels of adherence in each domain. Notably, 13% (4/30) of the apps (Parenting Plus, The Happy Child, Parenting Advice How to, and Parenting Solutions) only had information on communication or relationships or parental mental health but did not at all address tracking, supervision, positive reinforcement, consequences, clear rules, or effective requests.

**Table 5:** Domain-specific adherence (n=30).

Domains	Apps, n (%)	Level of adherence, mean (SD)
Relationship and communication	27 (90)	1.06 (0.49)
Praise	24 (80)	0.51 (0.42)
Parent mental health	23 (77)	1 (0.70)
Consequences	22 (73)	0.37 (0.30)
Psychoeducation	19 (63)	0.98 (0.73)
Behavioral targets or goals	19 (63)	0.41 (0.38)
Positive reinforcement	19 (63)	0.26 (0.27)
Clear rules	19 (63)	0.35 (0.35)
Maintenance or resources	19 (63)	0.42 (0.36)
Making requests	18 (60)	0.36 (0.30)
Tracking	10 (33)	0.16 (0.33)
Supervision	4 (13)	0.05 (0.15)

#### Overall Adherence Between Platforms

Across platforms, apps exclusive to the Apple App Store averaged 14.86% (SD 7%) on adherence level, apps exclusive to the Google Play Store averaged 22.76% (SD 7%), and those available on both stores averaged 21.37% (SD 12%).

#### Overall Adherence Based on App Popularity

Independent-sample 2-tailed *t* tests were used to determine whether there was a difference between apps with a user star rating of >3 and apps with low or no user ratings. There was no significant difference between the 57% (17/30) of highly rated apps evaluated for adherence (mean 20.32%, SD 12%) and the remaining 43% (13/30) of apps (mean 21.36%, SD 8%) in the level of adherence (*t*_29_=−0.25; *P=*.80).

Independent-sample 2-tailed *t* tests were used to determine whether there was a difference between the 4 most downloaded apps and the remaining apps. There was no significant difference between the most downloaded apps (mean 25.95%, SD 18%) and the remaining 90% (27/30) of the apps (mean 19.93%, SD 10%) in the level of adherence (*t*_29_=1.04; *P=*.31).

#### App Quality and Adherence

The Pearson correlation was calculated between the MARS overall score and overall adherence level*.* There was a significant positive correlation between the MARS score and adherence level (*r*_28_=0.4; *P*=.03), suggesting that, as the adherence score increased, the MARS rating also increased.

### Other Notable Apps and Features

#### Gamification

A notable app feature emerging from this review was gamification. A particularly notable example is an app named “Parent Hero,” which involves engaging cartoon or graphic stories of everyday scenarios. The app user is tasked with choosing responses at multiple points in a story, and their choices determine the plot that follows. At the start of each scenario, the app introduces the parenting skill to be practiced. For example, in the scenario “when a child doesn’t want to do something,” the user is first guided through cartoons that set the scene (“it’s almost time to take your child to kindergarten, but she still doesn’t have her shoes tied, so you said, ‘Katie, it’s time to go’”). Then, the user reaches a decisional point where they need to select a response from a list of possible options (“Boy, laces can really be difficult!”; “Come on, you’re a big girl. You know how to tie your shoes yourself”; and “We’re going to be late! Give me your foot!”). After selection of a response, the plot that follows the response is presented (eg, a quarrel gradually ensues following a selection of “We’re going to be late! Give me your foot!”). At the end of the story, informational pages are presented that discuss the reasons for the success or ineffectiveness of the selected response. The app contains a total of 17 unique scenarios categorized under 4 types of parenting skills—handling emotions (eg, “when a child wants something he cannot have”), engaging co-operation (eg, “when a child won’t clean up”), resolving conflicts (eg, “when children fight”), and praise and appreciation (eg, “when a child performs”). Thus, the parent can play with different responses in each story and learn responses that may be more effective in an entertaining way through the engaging stories. This app received consistently high MARS ratings across all subscales and overall scores (range 4-5; Multimedia Appendix 3). This indicates that the app is engaging while relatively well designed and functional.

Another approach to gamification is illustrated by the app “Parenting Challenge Quiz: 100+ Puzzles for Parents,” in which parents can take quizzes on a range of topics, from psychoeducation on child development to parenting skills. An example question from this app is “Rewards are something...: A. that are bought and given; B. need not be purchased; C. something that has high value.” Following selection of an answer, feedback is provided on whether the answer is correct or incorrect along with an expandable link to read further explanations. On the MARS, this app received acceptable overall and subscale ratings (from 3.4 on information to 4.75 on functionality; Multimedia Appendix 3). This app’s quiz-based nature helped its engagement compared with other text-based apps while maintaining high functionality (eg, performance and simplicity of navigation). However, its engagement may be improved, such as by incorporating images or videos within its quizzes or adding gamification features such as setting “accomplishments” or “goals” with answering quizzes.

#### Individualization

Although most apps (50/57, 88%) focused on general parenting skills with limited differentiation between child problem areas, some (7/57, 12%) targeted specific user populations and needs (Multimedia Appendix 2). Specifically, 9% (5/57) of the apps aimed to provide parenting tips for fathers (one of which had a counterpart for mothers, which included slightly different content). In addition, 4% (2/57) of the apps targeted a specific geographic location. For instance, “SMC Parenting for Dads” is an app that targets fathers in San Mateo County in California. Owing to its specificity, this app was able to include information about local resources, ideas for activities with children (eg, local playgrounds, hiking trails, and events), and even employment opportunities for fathers. However, compared with other apps, apps with these specifications did not appear to yield better MARS scores because of “acceptable” ratings on other MARS items, nor did they receive higher adherence ratings (Multimedia Appendices 3 and 4).

Parenting strategies differ across development. To guide future research, we also evaluated the extent to which the target age was considered as part of the app descriptions. Of the 57 apps, only 19 (33%) explicitly indicated an age range that the app was intended for either in the app store descriptions or screenshots, with 16 (28%) apps covering age ranges of ≤14 years and 8 (14%) covering an age range of ≥13 years (Multimedia Appendix 2). Furthermore, 67% (38/57) of apps that did not explicitly indicate an age range typically used language (eg, “kids” and “early development”) or examples (eg, tantrums) that suggested a focus on childhood concerns. The app quality and BPT adherence of age-specific apps did not appear to be significantly distinct from those of general apps, and the level of age-appropriate content was inconsistently (if not insufficiently) addressed across these apps. For example, the app “Raising Healthy Kids Age 6-17” provided content on developmental milestones, human papillomavirus vaccines, bullying, alcohol and drug use (vaping and kratom), sex, and resources for suicide. Although these topics are relevant for parents of teenagers, the content was broad and informational rather than specific and skill-based.

## Discussion

### Overview

This systematic review represents one of the first efforts to identify commercial parenting apps that include components of behavioral parenting techniques and to evaluate these apps on design quality (engagement, functionality, esthetics, and information) and adherence to strategies that are consistent with BPT interventions. The results of this review can be used to inform the development of behavioral parenting mHealth apps for parents of teenagers with behavioral problems given research that shows interest [16,17,58].

### App Characteristics

This review of commercial apps on 2 of the most widely used platforms (ie, Google Play Store and Apple App Store) revealed that parent-targeted mHealth apps with behavioral parenting components comprise a small percentage of available apps for “parents.” However, these apps were accessible. Most apps (49/57, 86%) were free to download, and only approximately one-fifth (10/49, 20%) of the free apps included in-app purchases.

The results also showed that 44% (25/57) of the apps were accessible on both app stores, 42% (24/57) were only accessible on the Google Play Store, and 14% (8/57) were exclusive to the Apple App Store. In addition, there were more downloads and user star ratings for the Google Play Store, suggesting higher engagement with apps on this platform. These results could be due to the larger number of people in the United States who own devices compatible with Google Play [59]. Nonetheless, these findings suggest that future app developers may prioritize releasing apps on the Google Play Store but should aim to release them on both platforms to maximize dissemination.

Consistent with previous literature on challenges and concerns regarding mHealth privacy [60,61], this study found that commercial apps dedicated an inconsistent and limited amount of attention to privacy. The locations of privacy policies varied across parenting apps, and some apps (17/57, 30%) did not include any information. Although it has been covered to a lesser degree in previous reviews of commercial apps, recent research suggests that privacy is an issue of particular concern for parents of adolescents with behavioral problems, and their teenage children have specific ideas about how privacy notices are to be displayed and made transparent (Ryan-Pettes, PhD, unpublished data, May 2023). Taken together, the results suggest that providers of parents should be aware of privacy concerns before recommending apps, and developers should be accountable for improving the accessibility (eg, privacy agreement in an easy-to-access location within the app) and transparency (eg, type of information shared and with whom the information may be shared) of privacy information [62-64].

### App Design Quality

Consistent with user star ratings between the Google Play and Apple App Stores, the MARS functionality ratings were similar across platforms. The MARS ratings also showed that apps accessible on both platforms had generally higher app design quality ratings compared with apps accessible only on the Google Play Store. These findings suggest that access to functional apps that are easy to use and simple to navigate appears to be approximately the same regardless of the app store used.

The finding that app quality was higher among apps on both the Apple App and Google Play Stores compared with those exclusive to the Google Play Store is consistent with previous mHealth literature [36,37]. An explanation is that apps meeting the release standards for both platforms also had more resources during development and design. Apps designed exclusively for the Google Play Store may require fewer resources given that the Apple App Store has stricter app release guidelines [65]. Despite apparently similar user star ratings for apps between these 2 platforms, the MARS scores suggest that apps released on both platforms have a higher design quality, which is related to user experience and engagement with the app [35]. Future developers of behavioral parenting intervention apps for parents should consider leveraging resources to meet the release criteria for both platforms or focusing on buttressing design quality by enhancing esthetics and engagement (eg, interactivity) before an exclusive release on the Google Play Store.

Importantly, the findings indicate that there is much room for improvement in the design quality of parenting apps. Average ratings of overall app quality as well as esthetics, information, and engagement were all within an “acceptable” range (rating of 3 on the MARS), whereas functionality appeared to be “good” on average (rating of 4 on the MARS). “Acceptable” quality ratings represent meeting the most basic criteria for design rather than an optimal or highly attractive design. Importantly, a substantial number of apps (21/51, 41%) evidenced minimal or simplistic designs (ie, a rating of 2 or below on interactivity on MARS), such as by using simple text-based presentation of information rather than high-quality designs that are adaptive and responsive. Given the importance of interactivity [66], future parent-targeted mHealth apps should focus on balancing high functionality with straightforward designs without sacrificing the features most relevant for engagement and behavior change [67]. For example, a future parent-targeted app may use minimalist yet professional and visually appealing color palates and straightforward navigation planes to preserve functionality. It may include user-friendly engaging features such as daily challenges that prompt parents to engage in specific effective parenting techniques (eg, praise their teenage child for a job well done and pause and take a deep breath before reacting to an upsetting child behavior).

Furthermore, the findings indicate that evaluation of app quality is important for an app’s commercial success. Consistent with the literature on user uptake and engagement, this study found that the most popular apps (higher user ratings and top downloads) received higher app quality scores on all domains of the MARS (ie, overall rating and all subscale ratings). In other words, the MARS ratings largely overlapped with user preference and popularity. Future development of parent-targeted mHealth apps for parenting may consider using established app quality rating scales such as the MARS to guide development.

### Adherence to Behavioral Parenting and Other Notable Features

In terms of adherence to different BPT domains, parental supervision (10/57, 18% of the apps) and tracking (4/57, 7% of the apps) were the 2 domains least addressed by the apps. These findings are unfortunate given the importance of these parenting skills for parents of adolescents with behavioral problems. However, encouragingly, the domain most addressed by the apps—family relationship and communication—also had the highest adherence scores.

An interpretation of these findings is that there may be more widespread interest in and higher demand for the relationship domain of parenting in mHealth apps. However, current apps do not cover communication strategies for issues that are common among parents of adolescents with behavioral problems (eg, delinquency, peer deviance, and substance use) [68]. Therefore, with this interpretation, the findings would suggest that parents of children with behavioral problems may still find current apps insufficient to meet all their needs and concerns. Indeed, previous research has shown that parents of children with substance use problems who are interested in using mHealth apps to support their parenting want additional parenting strategies related to monitoring, the implementation of consequences, and the initiation of positive activities with their teenage children in addition to communication skills [16]. Taken together, formative research with parents of children with behavioral problems is needed to help determine which additional BPT strategies should be included in a parent-targeted app for this population. As most developers do not use user-centered designs during commercial app development and instead evaluate the finished product, the limited existing formative research on commercial apps will likely not fill this gap [69,70].

A second interpretation of these findings is that there is a heavy focus on family relationships and communication as most apps (33/57, 58%) were designed for parents of young children or preadolescents. Research shows that building strong parent-child relationships is a central focus in childhood, and this shifts to monitoring and supervision during the teenage years [47]. With this interpretation, the results highlight an urgent need for research and development of apps for parents of teenagers or older children with behavioral problems. Focusing on this target population may help address gaps in access to evidence-based parenting support that is in high demand [3].

Importantly, across all parenting domains, the mean level of adherence to BPT principles and strategies was low among the included apps (mean 20.74%, SD 11% adherence to the strategies listed in our codebook). This finding suggests that most of the current commercial parenting apps do not sufficiently teach or approximate behavioral parenting techniques. Combined with results on low interactiveness, our findings suggest that commercial apps that are currently available to consumers generally underutilized the affordances of app technology to promote user engagement with the behavioral parenting components. First, a considerable number of apps (n=87) were excluded from this review as they served merely as aids or tools for enforcing parenting skills (eg, checklist of daily chores and a tracker for tokens) but did not provide didactic information on how to practice the underlying skills (eg, establishing house rules, reviewing house rules with the child, and consistently enforcing agreed-upon contingencies). Among the apps included in this study (all of which provide some informational instructions on parenting skills), many (21/51, 41%) present information in an unengaging and poorly adaptive and responsive way (eg, simple aggregation of texts taken from book chapters). Previous research suggests that interactivity with knowledge (consistent with the principles of social learning) is key such that parents can behaviorally practice and refine the skills [10]. Thus, current apps in the commercial market generally represent a suboptimal way to deliver BPT interventions or components digitally.

To improve adherence and enhance the effective delivery of behavioral parenting strategies, mHealth apps should integrate both didactic instructions and adaptive and responsive features consistent with social learning principles such that the underlying BPT skills can be learned and shaped into practice. As an illustration, the behavioral principle of consistency when implementing house rules (eg, a curfew of 9 PM) can be supported by app features such as daily push notifications at 8:50 PM to check the house for the teenager, and if the parent indicates “rule broken” on the app, it will be followed by automatic prompts for the parent to inform the teenager of this rule-breaking behavior and enforce the predetermined consequences right then (eg, decreased allowance). Insights from notable app features such as individualization and gamification can also be incorporated. For instance, parents may choose to personalize the time and frequency of prompts and receive individualized recommendations or examples of house rules based on the child’s age and the target problem behavior. The app can also include a progress tracker of the parent’s improvement over time and gamify the experience to boost engagement.

Despite low adherence, the apps included in this review showed a large number of downloads and average user ratings, suggesting good user satisfaction. Consistent with previous reviews [34], these findings suggest that parents’ willingness to try an app and their ratings of the app are not related to the degree to which the app includes intervention components that are scientifically grounded or implements parenting skills in a way that is related to increasing effective parenting. This study found that only 12% (6/51) of the apps received a rating of 5 on the “accuracy of app description” item on the MARS information subscale, suggesting that app descriptions on commercial app stores often do not provide a comprehensive view of the identity and expertise of app developers. Taken together, the results of this study add support for those calling for more regulation of health promotion mHealth apps in the commercial market, such as by mandating a description of the type of developer (eg, for profit) and expertise of the development team. The results also extend this call and recommend that app descriptions specify the extent to which the app features and content converge with the scientific basis.

This study found a medium positive correlation between overall app quality and overall level of BPT adherence. Although this result appears promising, it should be interpreted with caution. A reason is that the MARS inherently includes some measures of empirical support [35]. Specifically, the information subscale includes items such as “quality of information” (to what extent the overall app content is scientifically accurate and relevant), “credibility” (based on who the app developers are), and “evidence base” (whether there is empirical literature investigating this specific app; this item is scored as “n/a” and not counted toward the total score if no empirical literature was found). These 3 items may have some overlap with the app’s level of adherence to evidence-based parenting strategies, suggesting that the correlation found between app design quality (ie, MARS) and BPT adherence level may be overestimated in this study. However, these results are encouraging as they suggest the possibility of designing evidence-based behavioral parenting apps without sacrificing the features most relevant for engagement and behavior change. Examples include using simple (yet appealing) esthetics, easy-to-digest presentation, and personalization [67].

### Limitations

First, this review only included apps available in English in the US app stores and only looked at the 2 most popular commercial platforms. This excludes apps developed in other countries and languages from the scope of this review. There may also be English-language apps that are not currently found on either store that were not included in this review. However, surveying these 2 most dominant commercial platforms is consistent with previous research practices and adequately encompasses the dominant options for mHealth currently [37,38]. This study also excluded apps that were rated as <3 on the interactivity item in the MARS when adherence to behavioral parenting strategies and principles was assessed. It is possible that some of the excluded apps included more strategies and had higher adherence than the apps that were reviewed. However, our review of those with an interactivity of ≥3 showed that the commercial app industry is in the infancy stage of using effective app design and smartphone features to leverage the underlying BPT principles. Thus, it is highly unlikely that the excluded apps performed better. In addition, as discussed previously, they likely had poor user engagement because of low interactivity, limiting effectiveness for behavior change [53,66].

Second, given the lack of transparency in commercial app stores regarding information such as the specific number of downloads, it was difficult to obtain further empirical data on patterns or correlations related to the popularity of apps. Similarly, the number of reviews for an app may affect whether information on average user ratings was presented in the app store. In addition, user ratings may be highly variable and inconsistent, potentially raising questions about reliability [33,71]. Although this appears to be a common obstacle in reviews of commercial mHealth apps [36,37], it should be considered when interpreting the findings of this study.

Third, this study showed moderate levels of interrater reliability, particularly with regard to ratings of adherence to behavioral parenting techniques. Although the first author (KL) was the primary coder in this review, the BPT codebook was developed based on previous concerted efforts in the laboratory that involved a team of undergraduate students, 2 advanced doctoral students, and a clinical psychology faculty. The moderate level of reliability may be due to the differences in training and expertise between the 2 raters (KL and LD). Although the second rater (LD) was trained for 4 weeks before independently rating the apps, this rater was an undergraduate student in the laboratory who did not otherwise have coursework or experience in behavior theories or BPT.

Finally, this study aimed to inform the future design of mHealth apps for parents of adolescents. However, most of the apps identified (49/57, 86%) were either ambiguous regarding age range or focused on childhood (ages of <14 years). Although the review generated important insights for delivering behavioral parenting techniques via mHealth, the age range may be a key limitation when using currently available commercial apps to inform future apps for parents with adolescents.

### Conclusions

This study reviewed existing commercial apps for parenting skills and provided recommendations for future research. The 51 functional parenting apps identified across the Google Play and Apple App Stores largely fell short of providing BPT components in an adaptable, responsive, and engaging way, suggesting that current commercial apps still inadequately address BPT strategies in a way that is consistent with the underlying principles needed to increase the use of effective parenting strategies in the target population. This study found a moderate relationship between app quality and BPT adherence level and revealed that popular parenting apps appeared to have better app quality but not necessarily a higher level of adherence to evidence-based BPT strategies and principles. Findings from this review suggest that future app developers should consider novel, adaptive, responsive, and engaging ways to adapt traditional in-person behavioral parenting techniques to the mHealth format, such as gamification, individualization, and tailored content that is easy to digest and relatable to parents. Future researchers hoping to increase the dissemination of BPT-informed mHealth apps for parents should aim for free-to-download apps that are accessible on both platforms and balance high-quality design features (eg, simple esthetics, interactivity, and individualization) with content consistent with BPT principles. This may be accomplished through multisector (industry and academic) collaboration throughout the design process and involving end users (ie, parents) during different stages of app development.

## Appendix

Multimedia Appendix 1 Behavioral parenting core domains and examples.

Multimedia Appendix 2 Accessibility, popularity, and infrastructure parameters for each app.

Multimedia Appendix 3 Mobile App Rating Scale scores for each app.

Multimedia Appendix 4 Behavioral parenting adherence ratings for each app.

## Abbreviations

BPT: behavioral parenting training
ICC: intraclass correlation coefficient
MARS: Mobile App Rating Scale
mHealth: mobile health
