# Supplementary material for: Features, Design, and Adherence to Evidence-Based Behavioral Parenting Principles in Commercial mHealth Parenting Apps: Systematic Review
Source: JMIR Pediatr Parent. 2023 Jun 1;6:e43626. doi: 10.2196/43626 (PMC10273034; doi:10.2196/43626)
Supplement: Multimedia Appendix 1 [file pediatrics_v6i1e43626_app1.docx]

**Multimedia Appendix 1.** Behavioral Parenting Core Domains and Examples

| **Domain** | | | **Core Statements/Strategies** | | | | | | | | | |
| --- | --- | --- | --- | --- | --- | --- | --- | --- | --- | --- | --- | --- |
| Psychoeducation | | |  | | | | | | | | | |
|  | Information about normative child development. For example, what types of parental expectations, reinforcement options, or consequences are appropriate for the child's age. | | | | | | | | | | | |
|  | Information about problem/risk behaviors appropriate for child's age. For example, for parents of teenagers, information about warning signs of substance use and behavioral observations of intoxication. | | | | | | | | | | | |
| Setting Behavioral Targets | | | | | | |  | | | | | |
|  | Goals need to be achievable, realistic, developmentally appropriate, measurable, specific, and time limited | | | | | | | | | | | |
|  | Provides some guidance on selection of behaviors to target (or provide examples). | | | | | | | | | | | |
|  | It is important to define problem behaviors as well as their replacement! | | | | | | | | | | | |
|  | pinpointing: break down replacement behaviors into small steps | | | | | | | | | | | |
|  | Guidance on establishing and enforcing a behavior contract, including discussing with child in a positive tone, specifying responsibilities of the child, specifying responsibility of parent (monitoring and providing agreed upon rewards), reviewing the contract daily neutrally, and give rewards and praises immediately when earned. Do not take away rewards already earned. | | | | | | | | | | | |
|  | Guidance on establishing and enforcing a token economy, including setting behavioral goals, setting amount of tokens per each behavioral goal, setting a menu of possible rewards, setting the "price" of each reward, monitoring and tracking, and providing reinforcements or consequences immediately after behaviors. | | | | | | | | | | | |
| Tracking | | | | | | | |  | | | | |
|  | Defining behavior in a way that is specific, use objective/observable characteristics of behavior, clear, and as complete as you can (try to include all variations/range so as to prevent future debates about what counts or does not count). In other words, defining it so that anyone else (e.g., a babysitter, grandparent, older sibling) can tell whether the behavior has occurred. | | | | | | | | | | | |
|  | Assessing or knowing the baseline is important. | | | | | | | | | | | |
|  | Different ways to operationalize assessment of behavior may include: frequency in a period of time, ratio out of all possible opportunities, intensity, duration, latency, checklist (useful for chaining), whether it occurred in an interval, number of people who perform a behavior, using a checklist to track pinpointing | | | | | | | | | | | |
|  | It is important to track parent's own practice of parenting skills | | | | | | | | | | | |
|  | It is important to track house rules and provision of their consequences | | | | | | | | | | | |
|  | It is important to track behavior contract and its enforcement. | | | | | | | | | | | |
|  | It is important to track token economy routine and set up routine (e.g., weekly) time to review tokens and rewards. | | | | | | | | | | | |
|  | When tracking, parent needs to count and label behaviors in a specific and observable way. For instance, instruct parent to tell child what parent is doing, keep the tally/contract/rule posted, and as points are added or subtracted to the tally sheet, gently label (i.e., name explicitly) the behaviors as they occur, for both desirable and problem behaviors. | | | | | | | | | | | |
| Supervision | |  | | | | | | | | | | |
|  | Supervision involves answering four questions: who teen is with, where teen is, what teen is doing, when teen will be home. | | | | | | | | | | | |
|  | Networking with other adults (e.g., other parents) and getting to know teen's friends can be a powerful resource to gather information about the teen's whereabouts. | | | | | | | | | | | |
|  | Provide guidance on how to directly question your child, including both parenting behaviors to avoid (i.e., accuse, interrupt, use negative tone or sarcasm) and appropriate parenting behaviors (i.e., choose a good time, use neutral/positive tone, show interest/understanding, paraphrase/use reflection). | | | | | | | | | | | |
|  | Routine checkup. Every now and then, let your teen know that supervision and the relevant rules about teen's activities are because you care about her and her safety. Check to see that your teen is at the number or location she gave you. This also helps teach your teen accountability and to take your rules seriously. | | | | | | | | | | | |
|  | Street time or freedom (unsupervised time outside house, use of vehicles) should be clearly stated in house rules. App also provides examples or tips on determining what is the appropriate stress time for child's age (e.g., ask parents of a well-behaved peer of child's). | | | | | | | | | | | |
| Positive Reinforcement | | | |  | | | | | | | | |
|  | Emphasize contingent application: the reinforcer is provided when and only when the behavior occurs. | | | | | | | | | | | |
|  | Emphasize immediacy of delivery: there is little or no delay between the behavior and the reinforcer. This is especially important for younger kids, or when starting out the program. | | | | | | | | | | | |
|  | Emphasize schedule of reinforcement: At first continuous schedule (the behavior is followed by the reinforcer every time it occurs); overtime after behavior is developed, do not have to reinforce this behavior each time, but move on to a new behavior (so that total level of reinforcement is not decreasing overtime). | | | | | | | | | | | |
|  | Magnitude or amount of the reinforcer: The higher the magnitude (greater amounts) of a reinforcer delivered after the behavior, the more frequent the response will be (more effective), unless the reinforcer leads to satiation. High magnitude reinforcers recommended to be used during beginning of program. | | | | | | | | | | | |
|  | Quality or type of the reinforcer: High-quality reinforcers (e.g., preferred, those that an individual would engage in frequently or as much as possible) are more effective in changing behavior. Note that even the same reinforcer can vary in quality (e.g., praise, what type of tv a child watches when given tv time, satiation). | | | | | | | | | | | |
|  | A reinforcer is defined by its effects, rather than what is typically thought of as "rewards". Thus we need to ask and discuss with teen rather than assume.  In addition, we also need to be aware that teen's stated preference may or may not be high quality reinforcer. | | | | | | | | | | | |
|  | Provide information about two types of reinforcers: social and tangible. Give acceptable examples of both (e.g., praise, snacks, toys, movie, game, allowance, points or tokens). | | | | | | | | | | | |
|  | Selection of reinforcers: we don't want to limit a teen's access to necessities; reinforcers need to be extra stuff, not things that children should already ethically have access to (e.g., meals). It also should not be things we would object the child/teen having (e.g., alcohol, drugs, skip school). Need to be developmentally appropriate. | | | | | | | | | | | |
|  | When providing reinforcement, explicitly label what you are reinforcing them for | | | | | | | | | | | |
|  | Varied reinforcers: best to vary the reinforcers/rewards if possible, such as changing the praise or physical contact (pat, hug) when reinforcing. | | | | | | | | | | | |
|  | 1Combined reinforcers: Reinforcers combined, especially praise and tokens, can improve effectiveness over either one alone | | | | | | | | | | | |
|  | 1 Use of prompts: To be reinforced, the behavior must occur. To get the behavior to occur, prompts may be used in the form of modeling the behavior, providing verbal instructions, or actually helping to perform the task so the behavior can be reinforced. | | | | | | | | | | | |
|  | 1Practice trials. Ensure that there are many opportunities so the behavior or approximations of the behavior can be reinforced. If many opportunities do not occur, simulated situations can be carried out in the home. Reinforcement is provided for child behavior that the parent wishes to develop, even though this is a simulated or pretend situation. | | | | | | | | | | | |
|  | Reinforcement of each gradual step toward the final behavior. Do not wait for perfection to provide reinforcement. | | | | | | | | | | | |
|  | Reinforcement is better if it is small and frequent, rather than big and infrequent. Big and infrequent reinforcements may be reserved for completion of larger goals. | | | | | | | | | | | |
| Positive Verbal Support/Praise | | | | | | | | | | |  | |
|  | Rationale or any psychoeducation that may address potential misconceptions a parent may have about using praises, that may serve to increase parent's motivation to use praises. | | | | | | | | | | | |
|  | Make it simple and concise. Examples such as "great job doing xxx", "thank you for doing xxx". | | | | | | | | | | | |
|  | Emphasize immediacy. Praise should immediately follow positive/alternative behavior to the problem behavior | | | | | | | | | | | |
|  | Specifically label the behavior that parent is praising them on. | | | | | | | | | | | |
|  | The reinforcing quality of a praise can range in quality from high to low. Praise that sounds matter of fact or dismissive (e.g., saying “good” in a monotone) would be low in quality and distinguished from praise at the other end of the spectrum, which is delivered with enthusiasm, a positive facial expression, high volume, and physical contact such as touching and hugging (e.g., saying Great!! With all the nonverbal cues that might accompany that)." | | | | | | | | | | | |
|  | Guidance on how to deliver feedback (i.e., readily available, delivered immediately, provided in a metric that is meaningful in relation to some goal). | | | | | | | | | | | |
| Consequences | | | | | |  | | | | | | |
|  | Define "consequence" and give acceptable examples. For example, introducing that there are typically 4 types of consequences: corrective actions, loss of privileges, work chores, time-out. | | | | | | | | | | | |
|  | Selection of consequences: cannot be basic privileges (food, shelter, love) or growth privileges (prosocial development); need to be effective (actually experienced as aversive), and ethical. | | | | | | | | | | | |
|  | Guidance on what to say when giving a consequence (i.e., in a calm but firm tone of voice, label the problem behavior in terms of an established rule; state the consequence clearly; avoid arguing; ignore trivia). | | | | | | | | | | | |
|  | Provides some information preparing parents to manage their own emotional reactions when delivering consequences, so that parent can remain calm but firm. | | | | | | | | | | | |
|  | Provides information about what selective ignoring is, when it should or should not be used, and that it should be followed by praise as soon as problem behavior stops, or alternative behavior occurs. | | | | | | | | | | | |
|  | Guidance on how to manage more challenging problem behaviors (e.g., risky sex, substances, stealing/lying, truancy, safety), including 1) knowing the warning signs of these behaviors, 2) catching them early, not ignoring risky situations where more serious behavior could be learned and hidden, or wait for proof. When teen approaches parent and acknowledges the problem behavior, parent needs to acknowledge their effort to be honest without reacting emotionally or providing a severe consequence that may be harmful. | | | | | | | | | | | |
|  | Emphasize continued and consistent delivery, even if child provides a convincing explanation about the reasons for doing a problem behavior or breaking a rule | | | | | | | | | | | |
|  | Emphasize contingent delivery: punishment is delivered for the undesired behavior and not for other behaviors | | | | | | | | | | | |
|  | Emphasize immediacy of punishment. For instance, stating that even brief delays (e.g., 30 sec) can reduce effectiveness. | | | | | | | | | | | |
|  | Timing of punishment in the behavior sequence: the earlier, the more effective. | | | | | | | | | | | |
|  | Variation of punishment leads to greater suppression of the responses, and allows for using milder punishments | | | | | | | | | | | |
|  | Combined punishment should not be used in one instance - less practical/more difficult to implement, and unethical | | | | | | | | | | | |
|  | Punishment should be combined with reinforcement of alternative behavior | | | | | | | | | | | |
|  | It is important to consistently be mindful of and eliminate (as much as possible) any reinforcement for problem behavior. | | | | | | | | | | | |
|  | if mention use of time-out, define what time-out is (i.e., removal or loss of access to ideally all positive reinforcers that are normally available in the setting for a specified and brief period of time, e.g., less than 3 minutes). State that parent should give highly enthusiastic labeled praise after time-out ends to acknowledge child's compliance in time-out. | | | | | | | | | | | |
| Clear Rules/Setting Limits | | | | | | | | | |  | | |
|  | Provide guidance on steps to develop a list of house rules. Important steps include having both parent and child think of rules, bring these rules together to discuss with all members of the family, and at the end have at least one rule reflecting the wish of the child. | | | | | | | | | | | |
|  | Make sure rules as well as any consequences are agreed upon by all parties (all caregivers, parents, siblings), and are clearly and directly communicated | | | | | | | | | | | |
|  | Posting written rules and associated consequences in a place (e.g., refrigerator, digital app) so that everyone can see and monitor. | | | | | | | | | | | |
|  | Rules should be clearly stated, behaviors clearly defined, and expectations are appropriate and concrete. Should be stated in terms of positive behaviors (e.g., "Jim will be inside the house at 9pm on school nights"), rather than negative behaviors ("Jim will not be late"). | | | | | | | | | | | |
|  | Effectively monitor and track compliance of rules, even if not in parent's presence | | | | | | | | | | | |
|  | if a rule is violated more than once or twice, it may be necessary to add a consequence for breaking the rule. Before having the conversation with teen about consequences, go with a prepared list of consequences and prepare for a reaction from teen. | | | | | | | | | | | |
|  | Guidance on how to communicate the fact that parent is introducing new limits/consequences to child, including finding a good time to bring it up, being firm, and allowing for questions. | | | | | | | | | | | |
|  | House rules should be tailored to deal with current problems--remove ones that are no longer a problem and negotiate a new set of rules more appropriate to the age and sophistication of the child. | | | | | | | | | | | |
|  | Number of house rules: usually 5-10 | | | | | | | | | | | |
|  | Emphasize consistency in enforcing rules: rules should be enforced 100% of the time, across all caregivers/parents/relevant adults or individuals | | | | | | | | | | | |
|  | Give guidance on how to enforce a rule in a neutral manner. For instance, recommending that parent bring child to the posted rule and read the rule and its associated privilege/consequence out aloud. | | | | | | | | | | | |
| Making Requests | | | | | | | | |  | | | |
|  | Self-monitor and limit number of requests made by parent. | | | | | | | | | | | |
|  | Guidance on what to say when making requests: statements not questions; one request at a time; start-up requests rather than stop request; succinct; concrete and specific prompts (specific behaviors performed at specific times). | | | | | | | | | | | |
|  | Guidance on what not to do when making requests: questions, multiple requests at once, bringing up side issues / ancient history, blaming, criticizing, or showing disapproval. | | | | | | | | | | | |
|  | Guidance on nonverbals when making requests: a pleasant but firm tone; keep facial expression neutral; make eye contact; be polite and respectful | | | | | | | | | | | |
|  | Timing of requests: do not make requests while teen is in the middle of something; be aware of setting events or contexts that influence probability of compliance (e.g., teen's mood, physical fatigue) | | | | | | | | | | | |
|  | It can be helpful to use a series of high probability requests (high likelihood of compliance) before making a low probability request | | | | | | | | | | | |
|  | Giving child a clear definition of compliance, such as getting started on carrying out the request in 15 seconds the first time a request is made. | | | | | | | | | | | |
|  | Tracking compliance: if a request is made, a teen's response is either compliance or noncompliance. tell teen that you are keeping track of how often you are making requests and whether those requests are carried out. | | | | | | | | | | | |
|  | follow through a request with consistent, calm and predictable responses: when compliance, use labeled praise; when noncompliance, consider punishments (e.g., brief time out). never ignore any noncompliance. | | | | | | | | | | | |
| Relationship and Communication | | | | | | | | | | | |  |
|  | Active listening: attentive, warm, show understanding, nonjudgmental, paraphrase and ask if the understanding is correct, label child's emotions | | | | | | | | | | | |
|  | Parenting behaviors to avoid when being attentive: appear uninterested or inattentive, interrupt, interpret, accuse, think about child's intentions, blame, give advice, command | | | | | | | | | | | |
|  | Teaches any other positive communication components, such as: source responsibility ("I statement"), source directness (specific identification of "you" in expression), congruence (messages a congruent verbally, nonverbally, behaviorally and contextually), presentation of alternatives, impact statements (e.g., "when you do ___, the effect on me is ___"). | | | | | | | | | | | |
|  | Guidance on how to prep and discuss with child about specific sensitive topics (e.g., sex, substance use, divorce). | | | | | | | | | | | |
|  | Set aside routine and unobstructed time to talk (e.g., in car, doing errands/chores together, dinner, family meetings, having relaxed 15min day-to-day conversations). | | | | | | | | | | | |
|  | Teaches steps in effective negotiation or problem solving, which includes making neutral statements, brainstorming solutions, evaluating solutions, agree on selecting one solution, and setting time to follow-up to see how solution is working. Also includes guidance on how to negotiate effectively, including both things to do (e.g., finding appropriate time and place, warm and firm tone, remain neutral and objective, staying focused on the subject) and things to avoid (e.g., blaming, defending oneself, making broad generalizations, bringing up the past, lecture) | | | | | | | | | | | |
|  | Creating routine family time, where all members of family can engage in enjoyable recreational activity together. | | | | | | | | | | | |
| Parent Mental Health | | | | |  | | | | | | | |
|  | Address parent's confidence, self-efficacy or motivation in more than one way (e.g., encouragements and normalizing). | | | | | | | | | | | |
|  | Address parent's own mental health concerns in some way, such as by providing relaxation/self-care ideas, grounding techniques, mindfulness skills, or link to relevant external resources. | | | | | | | | | | | |
|  | Provides information on the importance of modeling; may be discussed in the context of specific problem areas (e.g., substance use). | | | | | | | | | | | |
| Maintenance and Resources | | | | | |  | | | | | | |
|  | Guidance on plan for maintenance (e.g. relapse planning). | | | | | | | | | | | |
|  | Provides crisis resources, including both crisis phone numbers and websites with links. | | | | | | | | | | | |
|  | Provides additional resources such as referrals to treatment services or links to websites and books. | | | | | | | | | | | |
