# Supplementary material for: Features, Design, and Adherence to Evidence-Based Behavioral Parenting Principles in Commercial mHealth Parenting Apps: Systematic Review
Source: JMIR Pediatr Parent. 2023 Jun 1;6:e43626. doi: 10.2196/43626 (PMC10273034; doi:10.2196/43626)
Supplement: Multimedia Appendix 2 [file pediatrics_v6i1e43626_app2.docx]

**Multimedia Appendix 2. Accessibility, Popularity, and Infrastructure Parameters for Each App**

| **App Name** | **Store** | **Cost** | **In-app Purchase/ Subscription** | **# of Downloads GP Only** | **# of Ratings GP** | **# of Ratings Apple** | **Mean Rating GP** | **Mean Rating Apple** | **Website** | **Privacy Policy Location** |
| --- | --- | --- | --- | --- | --- | --- | --- | --- | --- | --- |
| ADHD, Autism, Behavior problems | GP | $0 | $0.00 | 5000+ | 17 | - | 3 | - | No | None |
| Amira Parenting * | Both | $0 | $14.99-$79.99 | 1000+ | 8 | 9 | 4.5 | 5 | Yes | Website |
| Be a Better Father in 30 Days | Both | $0 | $0.99-$3.99 | 100+ | - | 2 | - | 4.5 | Yes | None |
| Be a Better Mother in 30 Days | Both | $0 | $0.99-$3.99 | 1000+ | 13 | 9 | 4.3 | 4 | Yes | None |
| BeDad: Parenting Tips for Dad | GP | $0 | $0.00 | 1000+ | 2 | - | 3 | - | Yes | App |
| Bee parenting | Both | $0 | $0.00 | 1000+ | - | - | - | - | Yes | None |
| Child behavior toolbox | A | $2.99 | $0.00 | - | - | - | - | - | Yes | None |
| Child Discipline Guide | GP | $0 | $0.00 | 100+ | - | - | - | - | No | None |
| Child toolbox - social skills | A | $2.99 | $0.00 | - | - | - | - | - | Yes | None |
| Dadditude: the happy dad app | Both | $0 | $4.99-$27.99 | 5000+ | - | 31 | - | 5 | Yes | Website |
| Developmental Parenting 6.0 | GP | $0 | $0.00 | 1000+ | - | - | - | - | Yes | None |
| Dr. Al's Parenting Tips & Tools | Both | $2.99 | $0.00 | 5+ | - | 3 | - | 5 | Yes | None |
| Excellent Parenting Tips | GP | $0 | $0.00 | 10+ | - | - | - | - | No | None |
| Good parenting skills | GP | $0 | $0.00 | 1000+ | - | - | - | - | No | Store |
| Guidepost parent | A | $0 | $0.00 | - | - | 25 | - | 4.5 | Yes | Store |
| Hire and Fire your Kids | Both | $0 | $0.00 | 5000+ | 61 | 9 | 2.9 | 4.4 | Yes | Store |
| How To Be A Good Dad - Tips And Advice | GP | $0 | $0.00 | 1000+ | - | - | - | - | Yes | None |
| How to Discipline Children Guide | GP | $0 | $0.00 | 10000+ | 33 | - | 5 | - | No | Store |
| How to discipline your kids | GP | $0 | $0.00 | 10000+ | 90 | - | 4 | - | No | Store |
| How to Improve Family Relationships Guide | GP | $0 | $0.00 | 100+ | - | - | - | - | No | Store |
| How to talk: parenting tips | Both | $0 | $0.00 | 5000+ | 29 | - | 3.6 | - | Yes | Store |
| Howtotalk: practical parenting | Both | $0 | $10.99-$349.99 | 5000+ | - | - | - | - | Yes | Website |
| It's what your children need (positive parenting) | GP | $0 | $0.00 | 5+ | - | - | - | - | Yes | Store |
| Kid - Adult Behavior Translator | GP | $0 | $0.00 | 1+ | - | - | - | - | No | None |
| Kids'Skills App | GP | $0 | $0.00 | 10000+ | - | - | - | - | Yes | None |
| Manatee: Mental health for families | Both | $0 | $0.00 | 1000+ | 14 | - | 4.3 | - | Yes | Store |
| NYS Parent Portal | Both | $0 | $0.00 | 50000+ | 86 | - | 4.3 | - | Yes | Store |
| Ommmm positive parenting | Both | $3.99 | $0.00 | 100+ | - | - | - | - | Yes | Website |
| Parent Lab - Parenting App for 0-12 | Both | $0 | $0.99-$249.99 | 100000+ | 847 | 241 | 4.1 | 4.6 | Yes | Store |
| Parent Parachute | GP | $0 | $0.00 | 1000+ | - | - | - | - | Yes | Store |
| Parenthing: parenting helpmate | A | $0 | $4.99-$9.99 | - | - | - | - | - | Yes | Store |
| Parenting - Advices For Parents | GP | $0 | $0.00 | 500+ | - | - | - | - | No | None |
| parenting\| advice\| how to | A | $0 | $0.00 | - | - | - | - | - | Yes | Store |
| Parenting Challenge Quiz: 100+ Puzzles for Parents | GP | $0 | $0.00 | 10000+ | 127 | - | 3.2 | - | Yes | Store |
| Parenting guide | GP | $0 | $0.00 | 5000+ | 24 | - | 5 | - | No | Store |
| Parenting Guidelines Tips | GP | $0 | $0.00 | 5000+ | - | - | - | - | Yes | Store |
| Parenting Hacks: Ultimate ideas, tips & quizzes | Both | $0 | $0.00 | 100+ | - | - | - | - | No | None |
| Parenting Healthy Kids Ages 6-17 | Both | $0 | $0.00 | 10+ | - | 3 | - | 5 | Yes | Store |
| Parenting Hero - Become a wiser parent | Both | $2.99 | $0.00 | 1000+ | 102 | 29 | 4.2 | 3.8 | Yes | Store |
| Parenting Skills | GP | $0 | $0.99-$3.99 | 5000+ | - | - | - | - | Yes | Store |
| Parenting solutions | Both | $1.99-$9.99 | $0.00 | 1+ | - | - | - | - | Yes | Store |
| Parenting Teens - The Gameplan | GP | $0 | $0.00 | 500+ | - | - | - | - | No | None |
| Parenting Tip | GP | $0 | $0.00 | 5000+ | - | - | - | - | No | Store |
| Parenting Tips - effective parenting information | GP | $0 | $0.00 | 5000+ | 18 | - | 4.3 | - | No | Store |
| ParentingNI | Both | $0 | $0.00 | 500+ | - | - | - | - | Yes | Store |
| Parenting Plus | A | $0.99 | $0.00 | - | - | 1 | - | 5 | Yes | Website |
| Positive Discipline | Both | $2.99 | $0.00 | 500+ | - | - | - | - | Yes | Store |
| Positive Parenting Tips | GP | $0 | $0.00 | 500+ | - | - | - | - | No | Store |
| Positivity Promoter | Both | $0 | $0.00 | 1+ | - | - | - | - | Link Broken | Link Broken |
| Smart Parenting \| Think-Grow Knowledge | Both | $0 | $1.99-$9.99 | 1000+ | - | 2 | - | 2 | No | Store |
| SMC parenting for dads | Both | $0 | $0.00 | 100+ | 8 | 4 | 5 | 5 | Yes | Store |
| Talk Share Learn Leeds | A | $0 | $0.00 | - | - | - | - | - | Yes | Store |
| The Happy Child | Both | $0 | $0.00 | 100000+ | 9892 | 734 | 4.9 | 4.9 | Yes | Store |
| Thumsters | Both | $0 | $1.99-$24.99 | 10000+ | 189 | 653 | 4.2 | 4.7 | Yes | Store |
| Top Child Care Tips | A | $0 | $0.00 | - | - | - | - | - | No | None |
| Weldon - Parenting Support (formerly Family Five) | Both | $0 | $0.00 | 100000+ | 520 | 82 | 4.5 | 4.9 | Yes | Store |
| WOW Parenting - Helping parents raise amazing kids | GP | $0 | $0.00 | 10000+ | 131 | - | 2.7 | - | Yes | Store |

Abbreviation: A, Apple; GP, Google Play
