# Supplementary material for: Features, Design, and Adherence to Evidence-Based Behavioral Parenting Principles in Commercial mHealth Parenting Apps: Systematic Review
Source: JMIR Pediatr Parent. 2023 Jun 1;6:e43626. doi: 10.2196/43626 (PMC10273034; doi:10.2196/43626)
Supplement: Multimedia Appendix 3 [file pediatrics_v6i1e43626_app3.docx]

**Multimedia Appendix 3.** Mobile App Rating Scale (MARS) Scores for Each App

| **App Name** | **Engagement** | **Functionality** | **Aesthetics** | **Information** | **Overall** |
| --- | --- | --- | --- | --- | --- |
| ADHD, Autism, Behavior problems | 1.6 | 4 | 2.00 | 3.40 | 2.75 |
| Amira Parenting | 4.4 | 4.5 | 5.00 | 4.50 | 4.60 |
| Be a Better Father in 30 Days | 3.4 | 4.5 | 4.00 | 3.40 | 3.83 |
| Be a Better Mother in 30 Days | 3.4 | 4.5 | 4.00 | 3.40 | 3.83 |
| BeDad: Parenting Tips for Dad | 3.8 | 4.75 | 4.00 | 2.60 | 3.79 |
| Child behavior toolbox | 3 | 4.75 | 3.00 | 3.80 | 3.64 |
| Child toolbox - social skills | 3 | 4.75 | 3.00 | 3.80 | 3.64 |
| Dadditude: the happy dad app | 4.2 | 4.5 | 4.67 | 3.80 | 4.29 |
| Developmental Parenting 6.0 | 2.6 | 4.25 | 3.00 | 2.80 | 3.16 |
| Dr. Al's Parenting Tips & Tools | 2.8 | 5 | 3.67 | 4.00 | 3.87 |
| Excellent Parenting tips | 2.8 | 5 | 4.00 | 2.80 | 3.65 |
| Good Parenting Skills | 2.8 | 5 | 3.00 | 3.80 | 3.65 |
| Guidepost parent | 3.8 | 4.75 | 4.33 | 3.60 | 4.12 |
| Hire and Fire your Kids | 4.6 | 4 | 4.33 | 4.20 | 4.28 |
| How To Be A Good Dad - Tips And Advice | 3.8 | 5 | 4.00 | 3.80 | 4.15 |
| How to Discipline Children Guide | 3.4 | 4.5 | 3.00 | 3.60 | 3.63 |
| How to discipline your kids | 3.6 | 4.5 | 2.67 | 3.60 | 3.59 |
| How to Improve Family Relationships Guide | 3.4 | 4.5 | 3.00 | 3.20 | 3.53 |
| How to talk: parenting tips | 3.6 | 4.25 | 4.67 | 4.20 | 4.18 |
| Howtotalk: practical parenting | 4.6 | 3.75 | 4.67 | 4.40 | 4.35 |
| It's what your children need (positive parenting) | 3 | 4.5 | 3.00 | 2.60 | 3.28 |
| Kids'Skills App | 3.4 | 4.5 | 4.00 | 4.40 | 4.08 |
| Manatee: Mental health for families | 4.6 | 4.75 | 5.00 | 4.67 | 4.75 |
| NYS Parent Portal | 3.6 | 3.75 | 4.00 | 3.83 | 3.80 |
| Ommmm positive parenting | 4 | 4.75 | 4.33 | 4.40 | 4.37 |
| Parent Lab - Parenting App for 0-12 | 4.8 | 4.75 | 5.00 | 4.67 | 4.80 |
| **App Name** | **Engagement** | **Functionality** | **Aesthetics** | **Information** | **Overall** |
| parent parachute | 4.4 | 5 | 5.00 | 4.50 | 4.73 |
| Parenthing: parenting helpmate | 4.2 | 4.75 | 4.33 | 4.40 | 4.42 |
| Parenting - Advices For Parents | 2.6 | 4.25 | 2.67 | 2.40 | 2.98 |
| Parenting \| advics \| how to | 3.8 | 4.5 | 3.67 | 3.80 | 3.94 |
| Parenting Challenge Quiz: 100+ Puzzles for Parents | 3.8 | 4.75 | 4.00 | 3.40 | 3.99 |
| Parenting guide | 2.6 | 4.5 | 3.67 | 3.20 | 3.49 |
| Parenting Guidelines Tips | 2.2 | 4.75 | 3.00 | 3.00 | 3.24 |
| Parenting Hacks: Ultimate ideas, tips & quizzes | 3.4 | 3.75 | 4.00 | 3.20 | 3.59 |
| Parenting Healthy Kids Ages 6-17 | 3.2 | 4 | 3.67 | 4.00 | 3.72 |
| Parenting Hero - Become a wiser parent | 4.4 | 5 | 4.67 | 4.00 | 4.52 |
| Parenting Skills | 3.2 | 3.75 | 3.00 | 3.00 | 3.24 |
| parenting solutions | 3.6 | 4.75 | 4.00 | 1.80 | 3.54 |
| Parenting Teens - The Gameplan | 3.2 | 4.75 | 3.67 | 3.80 | 3.85 |
| Parenting Tip | 2.2 | 4 | 2.33 | 2.80 | 2.83 |
| Parenting Tips - effective parenting information | 2.4 | 4.25 | 2.33 | 3.20 | 3.05 |
| ParentingNI | 3.2 | 4 | 4.00 | 3.60 | 3.70 |
| Parentingplus | 3.6 | 4.5 | 4.00 | 2.80 | 3.73 |
| Positive discipline | 4 | 5 | 4.67 | 4.00 | 4.42 |
| Positive Parenting Tips | 2.6 | 4.75 | 3.67 | 3.00 | 3.50 |
| Smart Parenting \| Think-Grow Knowledge | 3.4 | 4 | 3.00 | 3.80 | 3.55 |
| SMC parenting for dads | 3.4 | 4 | 3.67 | 4.00 | 3.77 |
| The Happy Child | 4.4 | 5 | 4.67 | 4.60 | 4.67 |
| Thumsters | 4.2 | 4.75 | 5.00 | 3.80 | 4.44 |
| Weldon - Parenting Support (formerly Family Five) | 4.2 | 5 | 4.33 | 4.00 | 4.38 |
| WOW Parenting - Helping parents raise amazing kids | 3.6 | 3.75 | 3.33 | 4.00 | 3.67 |
