# Supplementary material for: Features, Design, and Adherence to Evidence-Based Behavioral Parenting Principles in Commercial mHealth Parenting Apps: Systematic Review
Source: JMIR Pediatr Parent. 2023 Jun 1;6:e43626. doi: 10.2196/43626 (PMC10273034; doi:10.2196/43626)
Supplement: Multimedia Appendix 4 [file pediatrics_v6i1e43626_app4.docx]

**Multimedia Appendix 4. Behavioral Parenting Adherence Ratings for Each App**

| **App Name** | **Psychoeducation** | **Behavioral Target** | **Tracking** | **Supervision** | **Positive Reinforcement** | **Praise** | **Consequences** | **Clear Rules** | **Requests** | **Relationship and Communication** | **Parent Mental Health** | **Maintenance and Resources** | **Total** |
| --- | --- | --- | --- | --- | --- | --- | --- | --- | --- | --- | --- | --- | --- |
| Amira Parenting | 1.5 | 1.50 | 1.56 | 0 | 0.87 | 1.33 | 0.93 | 1.09 | 0.22 | 0.14 | 0.33 | 0.33 | 40.88% |
| Be a Better Father in 30 Days | 0 | 0.00 | 0.22 | 0 | 0.00 | 0.17 | 0.00 | 0.00 | 0.00 | 0.86 | 0.00 | 0.33 | 6.58% |
| Be a Better Mother in 30 Days | 0 | 0.17 | 0.00 | 0.2 | 0.00 | 0.17 | 0.00 | 0.00 | 0.00 | 0.86 | 0.00 | 0.33 | 7.18% |
| BeDad: Parenting Tips for Dad | 1.5 | 0.33 | 0.00 | 0 | 0.00 | 0.17 | 0.20 | 0.82 | 0.11 | 1.14 | 0.67 | 0.00 | 20.58% |
| Dadditude: the happy dad app | 0.5 | 0.00 | 0.00 | 0.2 | 0.00 | 0.33 | 0.33 | 0.18 | 0.00 | 1.14 | 2.00 | 0.67 | 22.33% |
| Guidepost parent | 1.5 | 0.00 | 0.00 | 0 | 0.20 | 0.33 | 0.00 | 0.00 | 0.56 | 0.86 | 1.67 | 0.67 | 24.08% |
| Hire and Fire your Kids | 0 | 0.50 | 0.67 | 0 | 0.33 | 0.33 | 0.07 | 0.45 | 0.11 | 0.00 | 0.00 | 0.00 | 10.27% |
| How to talk: parenting tips | 0 | 0.50 | 0.00 | 0 | 0.20 | 0.83 | 0.27 | 0.18 | 0.78 | 1.29 | 0.00 | 0.67 | 19.63% |
| Howtotalk: practical parenting | 0 | 1.00 | 0.22 | 0 | 0.47 | 1.50 | 0.40 | 1.09 | 0.56 | 1.43 | 1.67 | 0.00 | 34.71% |
| Manatee: Mental health for families | 0.5 | 0.83 | 0.56 | 0 | 0.47 | 0.00 | 0.13 | 0.27 | 0.67 | 1.43 | 0.67 | 0.33 | 24.40% |
| NYS Parent Portal | 1.5 | 0.00 | 0.00 | 0 | 0.00 | 0.17 | 0.00 | 0.36 | 0.00 | 0.86 | 0.67 | 1.00 | 18.98% |
| Ommmm positive parenting | 1.5 | 0.33 | 0.11 | 0 | 0.13 | 1.00 | 0.60 | 0.18 | 0.67 | 1.00 | 1.67 | 0.33 | 31.36% |
| Parent Lab - Parenting App for 0-12 | 1.5 | 0.83 | 0.33 | 0 | 0.20 | 0.00 | 1.13 | 1.09 | 0.78 | 1.86 | 2.00 | 1.00 | 44.69% |
| Parent Parachute | 1 | 0.50 | 0.00 | 0 | 0.33 | 0.17 | 0.27 | 0.18 | 0.56 | 1.00 | 1.33 | 0.67 | 25.02% |
| Parenthing: parenting helpmate | 1.5 | 0.00 | 0.00 | 0 | 0.07 | 0.00 | 0.60 | 0.00 | 0.22 | 0.57 | 0.33 | 0.33 | 15.11% |
| Parenting \| advics \| how to | 1.5 | 0.17 | 0.00 | 0 | 0.00 | 0.00 | 0.00 | 0.00 | 0.00 | 0.71 | 0.67 | 0.33 | 14.09% |
| Parenting Challenge Quiz: 100+ Puzzles for Parents | 1.5 | 0.50 | 0.11 | 0.2 | 1.07 | 0.67 | 0.40 | 0.36 | 0.33 | 0.86 | 2.00 | 0.00 | 33.33% |
| Parenting Hacks: Ultimate ideas, tips & quizzes | 1.5 | 0.17 | 0.00 | 0 | 0.53 | 0.67 | 0.47 | 0.00 | 0.44 | 1.57 | 1.67 | 0.33 | 30.62% |
| Parenting Healthy Kids Ages 6-17 | 2 | 0.33 | 0.00 | 0.8 | 0.00 | 0.33 | 0.13 | 0.18 | 0.00 | 0.86 | 0.00 | 1.33 | 24.88% |
| Parenting Hero - Become a wiser parent | 0 | 0.33 | 0.00 | 0 | 0.07 | 1.00 | 0.13 | 0.00 | 0.33 | 0.86 | 0.33 | 0.33 | 14.13% |
| Parenting Skills | 1 | 0.00 | 0.00 | 0 | 0.07 | 0.33 | 0.80 | 0.27 | 0.22 | 1.14 | 0.67 | 0.00 | 18.77% |
| Parenting Solutions | 1 | 0.00 | 0.00 | 0 | 0.00 | 0.00 | 0.00 | 0.00 | 0.00 | 0.00 | 0.00 | 0.33 | 5.56% |
| ParentingNI | 0.5 | 0.00 | 0.00 | 0 | 0.13 | 0.33 | 0.40 | 0.27 | 0.00 | 1.43 | 0.67 | 0.67 | 18.34% |
| Parentingplus | 0.5 | 0.00 | 0.00 | 0 | 0.00 | 0.17 | 0.00 | 0.00 | 0.00 | 0.14 | 0.67 | 0.00 | 6.15% |
| Positive Discipline | 0 | 0.33 | 0.00 | 0 | 0.20 | 1.00 | 0.67 | 0.73 | 0.56 | 1.43 | 1.33 | 0.00 | 26.02% |
| SMC parenting for dads | 0 | 0.00 | 0.00 | 0 | 0.00 | 0.00 | 0.20 | 0.09 | 0.00 | 0.57 | 1.33 | 0.00 | 9.15% |
| The Happy Child | 0 | 0.00 | 0.00 | 0 | 0.00 | 0.50 | 0.00 | 0.00 | 0.00 | 0.86 | 0.67 | 0.00 | 8.43% |
| Thumsters | 0 | 1.00 | 0.44 | 0 | 0.60 | 0.33 | 0.07 | 0.00 | 0.00 | 0.00 | 0.00 | 0.67 | 12.96% |
| Weldon - Parenting Support (formerly Family Five) | 2 | 0.50 | 0.44 | 0 | 0.33 | 1.00 | 0.33 | 0.45 | 0.89 | 1.43 | 1.67 | 0.00 | 37.71% |
| WOW Parenting - Helping parents raise amazing kids | 0 | 0.33 | 0.00 | 0 | 0.07 | 0.33 | 0.13 | 0.27 | 0.44 | 1.29 | 1.00 | 0.00 | 16.13% |
